# The Role of Computed Tomography-Determined Total Tumor Volume at Baseline in Predicting Outcomes of Patients with Locally Advanced Unresectable or Metastatic Pancreatic Ductal Adenocarcinoma

**DOI:** 10.3390/cancers18010020

**Published:** 2025-12-20

**Authors:** Elissar Moujaes, Jules Dupont, Littisha Lawrance, Fiona Frau, Ghina Jardali, Lama Dawi, Michèle Kind, Caroline Su, Samy Ammari, Nohad Masri, Anamaria Bianca Mihele, Valérie Boige, Thomas Pudlarz, Cristina Smolenschi, Marine Valéry, Geraldine M. Camilleri, Alice Boilève, Michel Ducreux, Nathalie Lassau, Antoine Hollebecque

**Affiliations:** 1 Department of Medical Oncology, Gustave Roussy Cancer Campus, 94805 Villejuif, Francevalerie.boige@gustaveroussy.fr (V.B.); marine.valery@gustaveroussy.fr (M.V.); antoine.hollebecque@gustaveroussy.fr (A.H.); 2 Department of Radiology, Gustave Roussy Cancer Campus, 94805 Villejuif, Francecaroline.su@aphp.fr (C.S.);; 3 Laboratoire d’Imagerie Biomédicale Multimodale Paris-Saclay (Biomaps) UMR1281, Institut National de la Santé et de la Recherche Médicale (INSERM), Centre National de la Recherche Scientifique (CNRS), Commissariat à l’Energie Atomique (CEA), Paris Saclay University, 94800 Villejuif, France; 4 Guerbet Research, 93420 Villepinte, France; 5 Department of Radiology, Bergonié Cancer Center, 33000 Bordeaux, France

**Keywords:** pancreatic ductal adenocarcinoma, metastatic, total tumor volume, prognosis, survival model

## Abstract

**Background:** Total tumor volume (TTV), derived from imaging data, has emerged as a potential prognostic biomarker in various cancers. This study aimed to evaluate the impact of TTV on outcomes in advanced pancreatic ductal adenocarcinoma (PDAC) and to validate a survival prediction model combining TTV with baseline clinico-biological markers. **Materials and Methods:** We conducted a retrospective analysis of 150 patients with locally advanced or metastatic PDAC treated with first-line FOLFIRINOX from 2010 to 2021. TTV was calculated by manually segmenting all visible lesions on baseline CT scans. Progression-free survival (PFS) and overall survival (OS) were the primary endpoints. A cut-off value for TTV predicting 6-month PFS was determined in 140 patients using AUC and Youden’s Index and then applied to OS analysis. A multivariate Cox regression model incorporating TTV, CA 19-9, and neutrophil-to-lymphocyte ratio (NLR) was developed in 94 patients to establish a survival risk score. **Results:** 12,028 lesions were annotated. OS was slightly but significantly different between TTV above and below the median value of 69.60 cm^3^ (12.4 vs. 13.5 months, *p* = 0.0269). A cut-off of 400 cm^3^ distinguished two groups: patients with TTV > 400 cm^3^ had significantly shorter OS (9.4 months) compared to those with TTV ≤ 400 cm^3^ (13.0 months, *p* = 0.0056). A similar trend was observed for PFS, though not statistically significant (7.4 months for TTV > 400 cm^3^ vs. 8.2 months for TTV ≤ 400 cm^3^, *p* = 0.0735). The combined model achieved a mean c-index of 0.62 for PFS and 0.64 for OS. Based on the risk score, high-risk patients had significantly worse median PFS (5.5 vs. 9.2 months, *p* = 0.0008) and median OS (7.2 vs. 13.5 months, *p* < 0.0001). **Conclusions:** TTV is a valuable prognostic marker in advanced PDAC. A model integrating TTV with biological markers enhances survival prediction and supports risk stratification in clinical practice.

## Simple Summary

Pancreatic adenocarcinoma remains one of the most aggressive cancers, and predicting prognosis and treatment response is a challenge. This study explores the utility of measuring the total tumor volume of primary and metastatic lesions on routine imaging scans for estimating survival in patients with advanced pancreatic cancer. We first analyzed patients who received standard chemotherapy and compared survival based on tumor volume and then developed a score that combines tumor volume with other biomarkers to predict survival. We found that patients with very large total tumor volumes have lower survival rates. When tumor volume was combined with tumor and inflammatory markers, the model provided a clearer way to distinguish patients at higher or lower risk of disease progression of death. These findings suggest that total tumor volume, together with biological markers, may guide treatment decisions in advanced pancreatic cancer.

## 1. Introduction

Despite recent therapeutics advances, pancreatic ductal adenocarcinoma (PDAC) remains the seventh leading cause of cancer death worldwide [1], with global incidence and mortality rates continuing to rise [2]. Standard treatment options for advanced PDAC and good performance status currently include FOLFIRINOX or Gemcitabine plus Nab-Paclitaxel, both of which have demonstrated superior survival outcomes compared to gemcitabine alone in large phase III trials [3,4]. 

The prognosis of PDAC depends on multiple clinical, biological and radiological parameters [5]. Among these, performance status at diagnosis remains the strongest clinical predictor of survival [6]. Additionally, baseline tumor markers, particularly CA 19-9, have been well established as significant prognostic factors [7,8]. In recent years, other inflammatory biomarkers such as C-Reactive Protein (CRP), serum Lactate dehydrogenase (LDH) and albumin, along with emerging hematologic ratios including neutrophil-to-lymphocyte ratio (NLR), monocyte-to-lymphocyte ratio (MLR) and platelet-to-lymphocyte ratio (PLR), have gained recognition for their prognostic relevance [9,10,11,12,13].

While several prognostic indices have been developed to predict survival outcomes in PDAC [14,15], most models do not integrate radiological characteristics. Although the RECIST criteria remain the gold standard for assessing treatment response during patient follow-up [16], they offer limited prognostic value at baseline evaluation. Consequently, total tumor volume (TTV) has emerged as a promising radiological marker for prognostication and survival prediction [17].

Recent studies have established a clear correlation between TTV and clinical outcomes across various cancers, particularly gastrointestinal malignancies. TTV has demonstrated prognostic value in predicting recurrence risk and survival after surgical resection in hepatocellular carcinoma and cholangiocarcinoma [18,19]. In colorectal cancer, baseline tumor volume has been shown to correlate with survival and treatment responsiveness, notably to immunotherapy [20]. Similarly, metabolic tumor volume assessed via positron emission tomography (PET) is predictive of survival in localized, resectable, or locally advanced PDAC [21,22]. Moreover, retrospective analyses have suggested an association between whole-liver tumor burden assessed by means of computed tomography (CT) and survival in metastatic PDAC with hepatic involvement [23,24].

However, the prognostic impact of baseline TTV specifically in advanced or metastatic PDAC treated with systemic chemotherapy has not yet been extensively evaluated. Given the significant variability in tumor burden among patients, accurately quantifying tumor volume at diagnosis could substantially enhance patient stratification and inform personalized therapeutic strategies.

Therefore, the primary objective of this study was to evaluate the prognostic significance of baseline TTV on progression-free survival (PFS) and overall survival (OS) in patients with locally advanced unresectable or metastatic PDAC receiving front-line FOLFIRINOX. Additionally, we sought to determine the prognostic value of integrating TTV with other clinico-biological biomarkers for improved survival prediction.

## 2. Materials and Methods

This retrospective study was conducted at a comprehensive cancer center (Institut Gustave Roussy), analyzing a large cohort of patients with histologically proven locally advanced or metastatic PDAC treated with front-line FOLFIRINOX or mFOLFIRINOX between 2010 and 2021. Patients who transitioned to a lighter chemotherapy regimen containing 5-Fluorouracil or Capecitabine were also included, whereas those initially treated with other first-line therapies were excluded to maintain cohort homogeneity.

**Figure 1:**
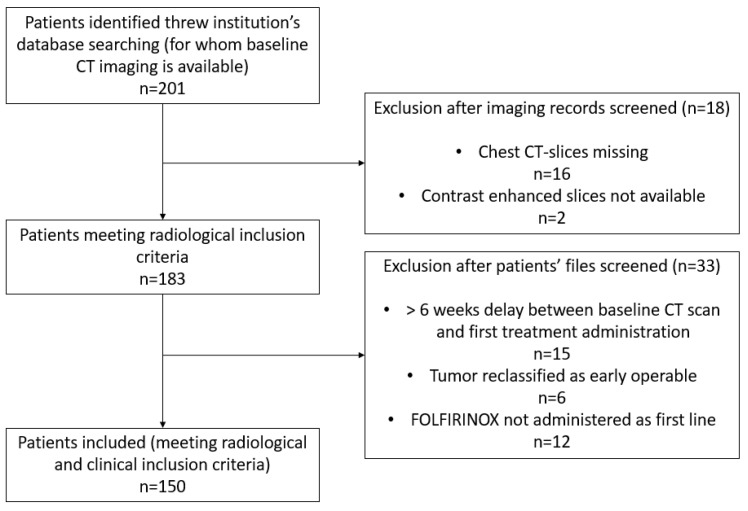
Flow chart illustrating the patient selection process. Abbreviations: CT = computed tomography; TTV = total tumor volume.

From an initial pool of 201 patients with baseline contrast-enhanced chest-abdomen-pelvis CT scans, 18 were excluded due to missing imaging data, and 15 due to initiation of chemotherapy more than six weeks after the baseline CT scan. Additionally, after a detailed review of clinical records, 6 patients were reclassified as having early-stage operable disease, and 12 were identified as having received FOLFIRINOX in a later line of therapy. Ultimately, 150 patients fulfilled all inclusion criteria and were retained for analysis. The patient selection flowchart is detailed in Figure 1.

### 2.1. Data Collection

Data were collected retrospectively from patient medical records, supplemented by the national death registry published by the Institut National de la Statistique et des Etudes Economiques (INSEE) to obtain missing survival information. Imaging data were extracted directly from the institutional imaging software.

### 2.2. Imaging Analysis

**Figure 2:**
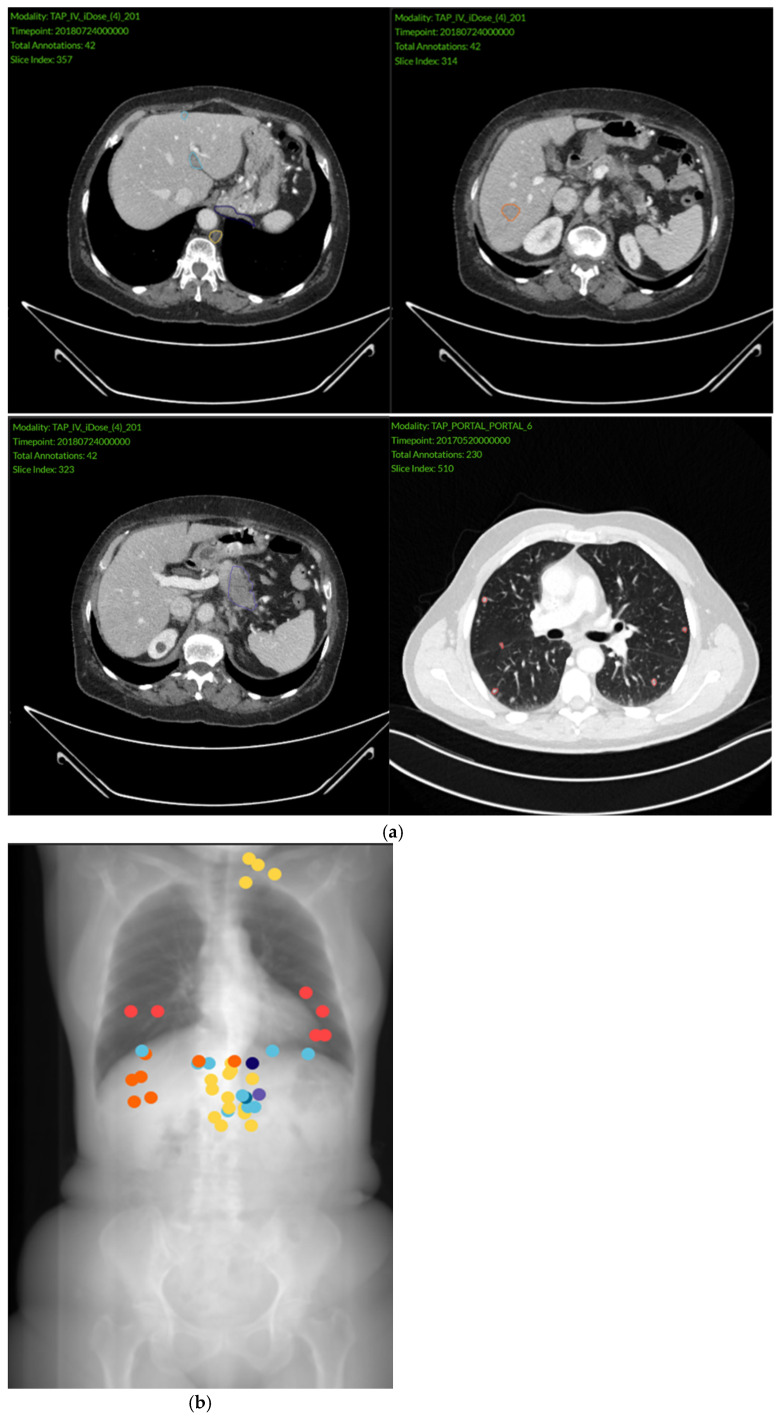
Examples of lesion annotations. (**a**) An example of annotations of primary and metastatic lesions on CT scan axial slices. Violet = pancreatic tumor, yellow = lymph node metastasis, blue = peritoneal carcinomatosis, light orange = liver metastasis, dark orange = lung metastasis; (**b**) an example of a scout view of a chest-abdomen-pelvis CT scan with all the annotations labeled by colors according to the tumor location. Abbreviations: CT = computed tomography.

Contrast-enhanced chest-abdomen-pelvis CT scans were analyzed by four experienced independent radiologists after anonymization. All visible primary and metastatic lesions were manually circumscribed in 2D at their largest diameter with an in-house software (Figure 2a). Segmented lesions were individually labeled according to their organ or structure of location (Figure 2b). Approximate tumor volume was obtained for each lesion using the following formula: Tumor volume = 2/3 × Surface × Minor Axis, and summed per patient to obtain TTV. This simplified formula was selected for its efficiency and feasibility in routine practice, as fully three-dimensional volumetric segmentation, although potentially more accurate, remains more time-consuming and technically demanding. The surface and minor axis were computed using Pyradiomics 3.0.1. 10 patients for whom TTV calculation could not be achieved were excluded from further descriptive and predictive analyses.

### 2.3. Baseline Parameters and Outcomes

Our primary objective was to evaluate the prognostic impact of baseline TTV on PFS and OS in patients receiving front-line FOLFIRINOX. A secondary objective was to develop a predictive model integrating TTV with other clinic-biological markers to predict survival outcomes. OS and PFS served as the endpoints for these analyses.

### 2.4. Statistical Analysis

#### 2.4.1. Descriptive Analysis

The distribution of continuous variables is expressed in median and IQR (Interquartile range).

All analyses were performed with Python (v3.9.19) using a 5% significance level. 

We used a bootstrap-based AUC analysis with 10,000 iterations to assess the performance of TTV in distinguishing patients with PFS or OS above vs. below 6 months. We compared PFS and OS between patients with TTV above or below the median, then used Youden’s Index to find a common TTV threshold for both endpoints. A log-rank test with Bonferroni correction was applied to compare survival in the subgroups determined by these thresholds.

A Spearman correlation matrix was created to assess the relationships among TTV, CA 19-9, NLR, and MLR—all chosen for their reported prognostic value. Patients missing any of these parameters were excluded, leaving 94 in the final analysis.

Univariate and multivariate Cox regression analysis were performed on baseline parameters for PFS and OS after binarization as follows:

- For TTV, the cut-off established in the initial analysis was adopted.
- The cut-off of 1000 U/L was used for CA 19-9 based on data from the literature [25,26,27].
- The cut-off of 5 was used for NLR based on data from the literature [28].

#### 2.4.2. Predictive Analysis

The primary objective was to create a model that defines a risk score that combines TTV with biological parameters, using previously detailed cut-offs. A Cox regression model was fit on baseline data for PFS and OS. The generalization performance was assessed through 10-fold cross-validation. A risk score was defined as the sum of the Cox model’s coefficients multiplied by the binary values of the variables. We then used the median score to split patients into high- and low-risk groups, comparing survival with Kaplan–Meier estimators. 

All data were blinded to the statistician and the radiologist performing the segmentation.

## 3. Results

### 3.1. Characteristics of the General Population

Our population was well-balanced regarding gender, consisting of 48% females and 52% males, with a median age at diagnosis of 60 years (IQR = 16 years). Among the 150 patients, only nine presented with locally advanced unresectable disease, while the remaining had metastatic disease, with 83.7% being de novo metastatic and 16.3% secondary metastatic. 90.7% of the patients exhibited a good performance status (ECOG 0-1).

Demographic and clinical characteristics at baseline are detailed in Table 1. Approximately one-half of the patients had elevated CA 19-9 (>1000 U/L) and roughly 40% had increased Carcinoembryonic Antigen (CEA) values. The LMR was elevated in more than half of the patients, whereas a high NLR ratio (NLR ≥ 5) was observed in only about one quarter of patients with available data. Distribution of tumor and inflammatory markers is detailed in Table 2.

### 3.2. Imaging Findings

**Figure 3:**
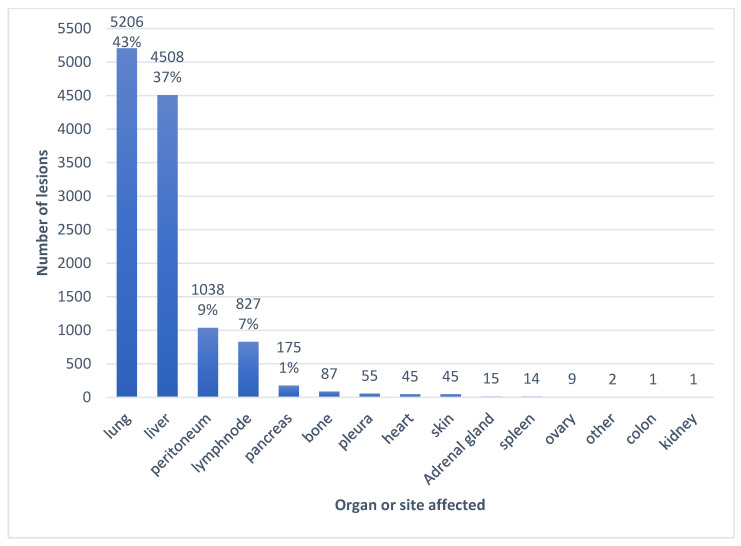
Distribution of lesions according to organs or sites.

In 140 patients, 12,028 primary and metastatic lesions were circumscribed. The most frequently involved metastatic sites were the lungs with 5206 lesions, followed by the liver with 4508 lesions. The distribution of lesions according to affected organs is illustrated in Figure 3. On average, patients had lesions in six different organs (including the pancreas).

The median TTV in our cohort was 69.60 cm^3,^ and its distribution across the patient population is illustrated in Supplementary Material (Figure S1). 

### 3.3. Patient Survival According to TTV

The area under the curve (AUC) analysis conducted at 6 months for TTV on 140 patients was 0.49 (95% CI: 0.39–0.60) for PFS and 0.72 (95% CI: 0.57–0.82) for OS (Supplementary Material; Figure S2). These results suggest that TTV has a limited predictive value for PFS but exhibits a moderate association with OS.

When comparing PFS and OS using Kaplan–Meier estimators for patients divided by the median TTV, no significant difference was observed in PFS (7.5 months for TTV below median vs. 7.9 months for TTV above median, *p* = 0.675). However, a modest yet statistically significant difference was found in OS (median OS: 13.5 months for TTV below median vs. 12.4 months for TTV above median, *p* = 0.027).

To establish a clinically meaningful TTV threshold for predicting differences in PFS and OS, we performed additional analysis using Youden’s Index based on the AUC results. This approach identified a TTV cut-off of 384 cm^3^, which we rounded to 400 cm^3^ for clearer clinical applicability (Supplementary Material; Figure S3).

Patients were subsequently divided into two groups: those with TTV > 400 cm^3^ and those with TTV ≤ 400 cm^3^. Patients with TTV > 400 cm^3^ exhibited shorter median PFS (7.4 months) compared to those with TTV ≤ 400 cm^3^ (8.2 months). This difference was initially statistically significant (*p* = 0.0368) but approached significance after Bonferroni correction (*p* = 0.0735). Importantly, the 400 cm^3^ cut-off clearly separated the groups regarding OS, with median survival significantly shorter for patients above the threshold compared to those below it (9.4 months vs. 13.0 months; *p* = 0.0057).

**Figure 4:**
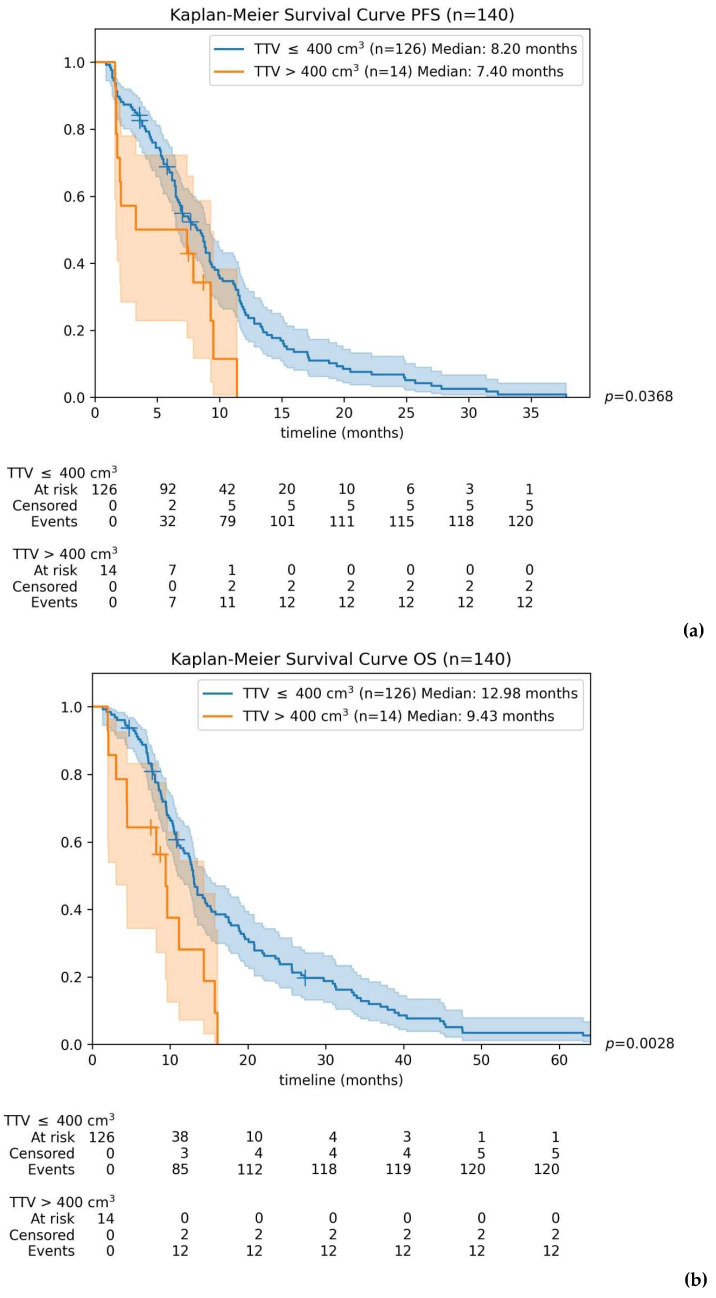
Kaplan–Meier curves for progression-free survival (**a**) and overall survival (**b**) according to the determined cut-off of total tumor volume (TTV). *p*-values are presented prior to Bonferroni correction. Median PFS is 7.4 months in patients with TTV > 400 cm^3^ vs. 8.2 months for TTV ≤ 400 cm^3^ (*p* = 0.0368). Median OS is 9.4 months in patients with TTV > 400 cm^3^ vs. 13.0 months for TTV ≤ 400 cm^3^ (*p* = 0.0057).

Kaplan–Meier curves illustrating differences in PFS and OS according to the established TTV threshold of 400 cm^3^ are presented in Figure 4.

### 3.4. Correlation Between Baseline Parameters

**Figure 5:**
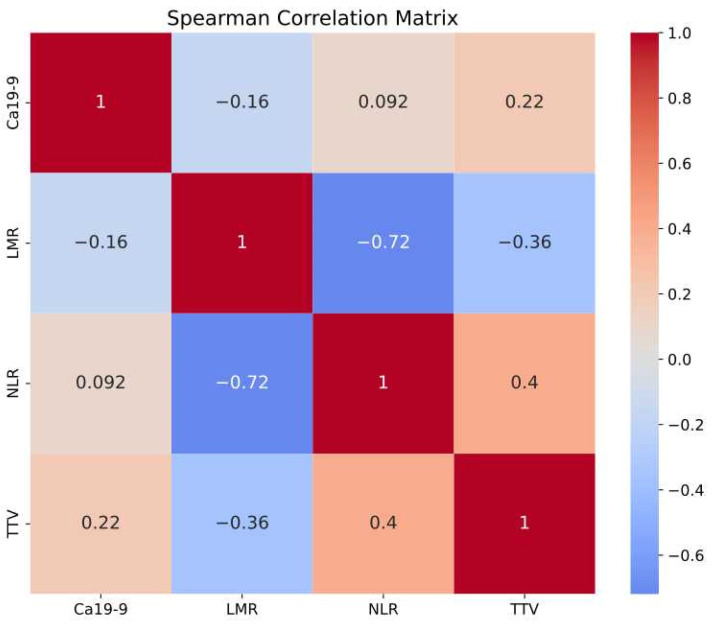
Spearman correlation matrix for baseline parameters.

The Spearman Correlation Matrix presented in Figure 5 demonstrates a strong correlation between NLR and LMR. Additionally, both MLR and NLR showed moderate associations with TTV, whereas CA 19-9 exhibited only a weak correlation with TTV.

### 3.5. Survival Model According to a Combined Risk Score

Our objective was to develop a predictive survival model for PDAC patients by integrating TTV with additional prognostic parameters. Based on previous findings, we selected CA 19-9 as a tumor marker and NLR as an inflammatory marker. Given its strong correlation with NLR and the limited existing literature regarding its prognostic value in PDAC, MLR was excluded from this predictive model. This analysis included data from 94 patients.

We applied Cox regression modeling incorporating TTV, CA19-9 and NLR to predict PFS and OS, generating a risk score capable of stratifying patients into distinct survival groups based on predefined thresholds.

In univariate analysis, only NLR demonstrated a statistically significant Hazard Ratio (HR) for PFS and OS. In multivariate analysis, NLR retained its significance for both PFS and OS, while CA 19-9 approached statistical significance for OS. Detailed results from these univariate and multivariate analyses are provided in Supplementary Material (Table S1).

The predictive model’s generalization capability was evaluated using a 10-fold cross validation. There was no statistically significant difference between training and testing sets (*p* = 0.665 for PFS, *p* = 0.955 for OS), indicating robust model generalization.

For PFS, the model achieved a mean c-index of 0.605, (95% CI: 0.550–0.660) on the test folds with an optimal risk-score cut-off set at 0.30.

Regarding OS, the mean c-index achieved was 0.645 (95% CI: 0.604–0.686) with a risk-score threshold of 0.38.

**Figure 6:**
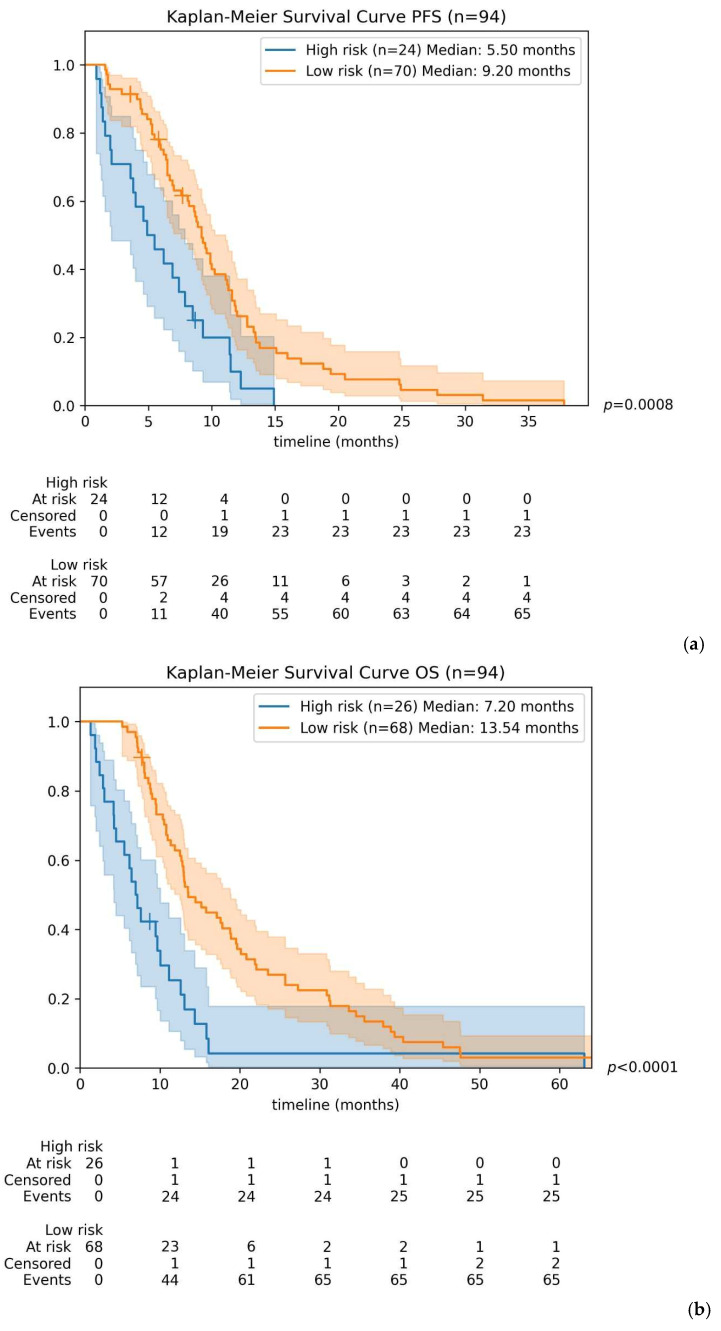
Kaplan–Meier curves for progression-free survival (**a**) and overall survival (**b**) according to defined risk score in the population. Median PFS is 5.50 months in patients with a risk score above the cutoff vs. 9.20 months in patients with a lower risk score (*p* = 0.0008). Median OS is 7.20 months in patients with a risk score above the cutoff vs. 13.50 months in patients with a lower risk score (*p* < 0.0001).

Patients identified as high-risk had significantly shorter median survival compared to low-risk patients: 5.50 vs. 9.20 months for PFS (*p* = 0.0008), and 7.2 vs. 13.5 months for OS (*p* < 0.0001). Kaplan–Meier curves illustrating these differences in PFS and OS for high-risk versus low-risk groups are presented in Figure 6.

## 4. Discussion

In this cohort of patients with advanced PDAC, demographics and clinico-biological characteristics of patients closely matched those reported in large phase III trials [3].

To our knowledge, this is the first study to establish a TTV threshold predictive of survival outcomes in advanced PDAC patients. Our analysis identified a median TTV of 69.60 cm^3^, whereas the optimal survival threshold determined was notably higher at 400 cm^3^, over five times greater than the median. This substantial difference underscores that TTV is particularly useful in identifying patients with an exceptionally high tumor burden.

For context, this 400 cm^3^ threshold represents roughly 25% of the average human liver volume, estimated at about 1500 cm^3^, highlighting its clinical significance. Patients exceeding this threshold are therefore at substantially increased risk for rapid disease progression and reduced survival due to their extensive tumor load.

Previous studies in pancreatic cancer have demonstrated an inverse correlation between tumor volume and survival in localized or locally advanced disease, often incorporating TTV alongside other biomarkers into combined predictive models [29,30,31]. It is also well established that PDAC patients with isolated lung metastasis generally have better prognoses than those with liver metastases. The number of metastatic sites also plays a role in determining prognosis in PDAC [32]. In our cohort, the lungs were the most frequently affected metastatic site, surpassing the liver. This observation emphasizes the importance of specifically considering pulmonary metastases in analyses evaluating the prognostic impact of TTV.

The emerging field of radiogenomics enables the prediction of tumor phenotypes and behavior based on integrated genomic and radiomic signatures [33,34]. Furthermore, circulating tumor DNA (ct-DNA) levels have been correlated with tumor volume in both metastatic and non-metastatic pancreatic cancer settings, and both markers have been associated with survival outcomes [35,36]. We hypothesize that patients with advanced pancreatic cancer characterized by high tumor volume likely exhibit higher clonal heterogeneity, rendering them more prone to developing treatment-resistant sub-clones during chemotherapy course and thereby accelerating disease progression.

Our study, however, has several limitations inherent to its retrospective, monocentric and observational design. Firstly, tumor volume was estimated using a simplified two-dimensional measurement formula instead of fully three-dimensional volumetric contouring, which, although potentially more accurate, is significantly more time-consuming [37]. Besides, our choice of the 2D approximation formula was dictated by the in-house developed software. This calculation method assumes a near-spherical geometry and tends to overestimate volumes, and is not the classical gold standard reported in the literature for accurate volumetry, nor is it validated against standard volume estimations.

Additionally, lesion tagging was performed by four different radiologists, potentially introducing inter-observer variability. Importantly, the absence of a validation cohort limits our ability to fully generalize the results.

We acknowledge that missing data represent an important limitation of this study. Multiple imputation could have been used to reduce potential estimation bias associated with missing values for CA 19-9 and NLR; however, this analysis was not performed because assessment of the prognostic value of these variables was not a primary objective of the study. With regard to missing TTV values, they accounted for less than 10% of the dataset and were assumed to be randomly distributed; therefore, we believe they are unlikely to have affected the interpretation of our results.

Moreover, patients who underwent treatment de-escalation were included without specific stratification or subgroup analysis, which might have influenced survival outcomes. Of note, this study only included patients who received FOLFIRINOX or mFOLFIRINOX in the first-line setting to ensure cohort homogeneity. However, many patients in real life are not fit for this scheme and receive other regimens such as gemcitabine in monotherapy or in combination with nab-paclitaxel. Including these patients in future studies would be interesting to assess the prognostic value of TTV in this population.

Our analysis allowed us to create a survival model that demonstrates a modest, but real, discriminatory ability to distinguish PDAC patients with a high risk of progression or death. However, this tool should be used with caution for individual-level decision-making in clinical practice. Of note, although being one of the most well-established prognostic factors in PDAC, we believe that including ECOG performance status in the predictive model would add limited discriminatory value, as the majority of patients in our cohort had a good performance status.

Considering FOLFIRINOX remains the standard-of-care first-line therapy, future research efforts should prioritize validating predictive models and optimizing therapeutic strategies specifically tailored for this high-risk patient subgroup.

## 5. Conclusions

Our study highlights the prognostic significance of very high total tumor volume (TTV) in patients with locally advanced or metastatic PDAC. Notably, TTV demonstrated a stronger predictive association with overall survival than progression-free survival, particularly for values significantly above the median. Patients exhibiting a very high TTV experienced both rapid progression following first-line FOLFIRINOX and significantly reduced overall survival.

These findings highlight the potential utility of an integrated predictive model combining radiological and biological markers, which could enable more accurate survival forecasting and facilitate personalized therapeutic approaches for high-risk PDAC patients.

## Figures and Tables

**Table 1:** Demographic and clinico-biological characteristics of patients at baseline.

Characteristic	N = 150
Age (year)	
Median	60
IQR	16
Sex—*n* (%)	
Male	78 (48%)
Female	72 (52%)
BMI (kg/m^2^)	
Median	24
IQR	5.7
Alcohol consumption—*n* (%)	
Yes	38 (25.4%)
No	83 (55.3%)
NA	29 (19.3%)
Smoking status—*n* (%)	
Smoker	64 (42.7%)
Non-smoker	60 (40%)
NA	26 (17.3%)
ECOG Performance status score—*n* (%)	
0	74 (49.3%)
1	62 (41.4%)
2	8 (5.3%)
3	1 (0.7%)
NA	5 (3.3%)
Pancreatic tumor location—*n* (%)	
Head	44 (29.3%)
Uncus	15 (10%)
Body	4 (2.7%)
Isthmus	27 (18%)
Tail	31 (20.7%)
Multicentric	29 (19.3%)

**Table 2:** Tumor markers and inflammation markers at baseline.

Characteristic	N = 150
Ca 19-9—*n* (%)	
<37 U/L	29 (19.3%)
37–1000 U/L	34 (22.7%)
≥1000 U/L	73 (48.7%)
NA	14 (9.3%)
Median	2064
IQR	7789
CEA—*n* (%)	
<5	75 (50%)
≥5	59 (39.3%)
NA	16 (10.7%)
Median	40.2
IQR	133.6
Neutrophil-to-Lymphocyte Ratio—*n* (%)	
<5	87 (58%)
≥5	37 (24.7%)
NA	26 (17.3%)
Median	3.6
IQR	2.9
Lymphocyte-to-Monocyte Ratio—*n* (%)	
<2	43 (28.7%)
≥2	81 (54%)
NA	26 (17.3%)
Median	2.3
IQR	1.8

## Data Availability

Dataset available on request from the authors.

## Supplementary Materials

The following supporting information can be downloaded at https://www.mdpi.com/article/10.3390/cancers18010020/s1. Figure S1: Distribution of TTV in the overall population; Figure S2: AUC analysis of TTV for PFS and OS at 6 months; Figure S3: Kaplan–Meier curves for progression-free survival (A) and overall survival (B) according to median total tumor volume (TTV); Table S1: Univariate (A) and multivariate (B) analysis on baseline parameters for PFS and OS.

## Abbreviations

The following abbreviations are used in this manuscript:

AUC	Area under the curve
BMI	Body mass index
CA 19-9	Carbohydrate antigen 19-9
CEA	Carcinoembryonic antigen
CRP	C-Reactive Protein
CT	Computed tomography
DNA	Deoxyribonucleic acid
ECOG	Eastern Cooperative Oncology Group
INSEE	Institut National de la Statistique et des Etudes Economiques
IQR	Interquartile range
LDH	Lactate dehydrogenase
MLR	Monocyte-to-lymphocyte ratio
NLR	Neutrophil-to-lymphocyte ratio
OS	Overall survival
PDAC	Pancreatic ductal adenocarcinoma
PET	Positron emission tomography
PFS	Progression free survival
PLR	Platelet-to-lymphocyte ratio
RECIST	Response evaluation criteria in solid tumors
TTV	Total tumor volume
