# Supplementary material for: The Role of Computed Tomography-Determined Total Tumor Volume at Baseline in Predicting Outcomes of Patients with Locally Advanced Unresectable or Metastatic Pancreatic Ductal Adenocarcinoma
Source: Cancers (Basel). 2025 Dec 20;18(1):20. doi: 10.3390/cancers18010020 (PMC12784911; doi:10.3390/cancers18010020)
Supplement: Supplementary file 1 [file cancers-18-00020-s001.zip › cancers-4013152-supplementary.pdf]

Supplementary material

	TTV
count	140.000000
mean	183.774910
std	335.964582
min	0.736047
25%	28.355394
50%	69.597605
75%	171.019827

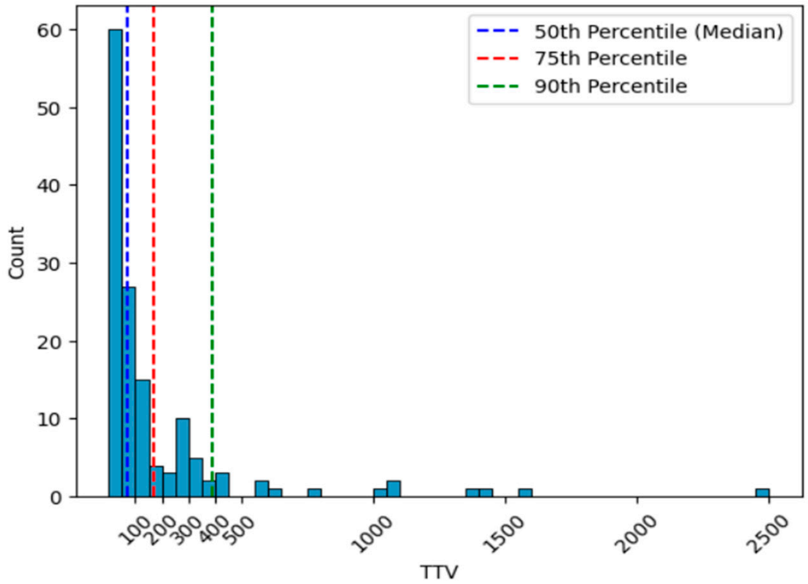

Figure S1 : Distribution of TTV in the overall population

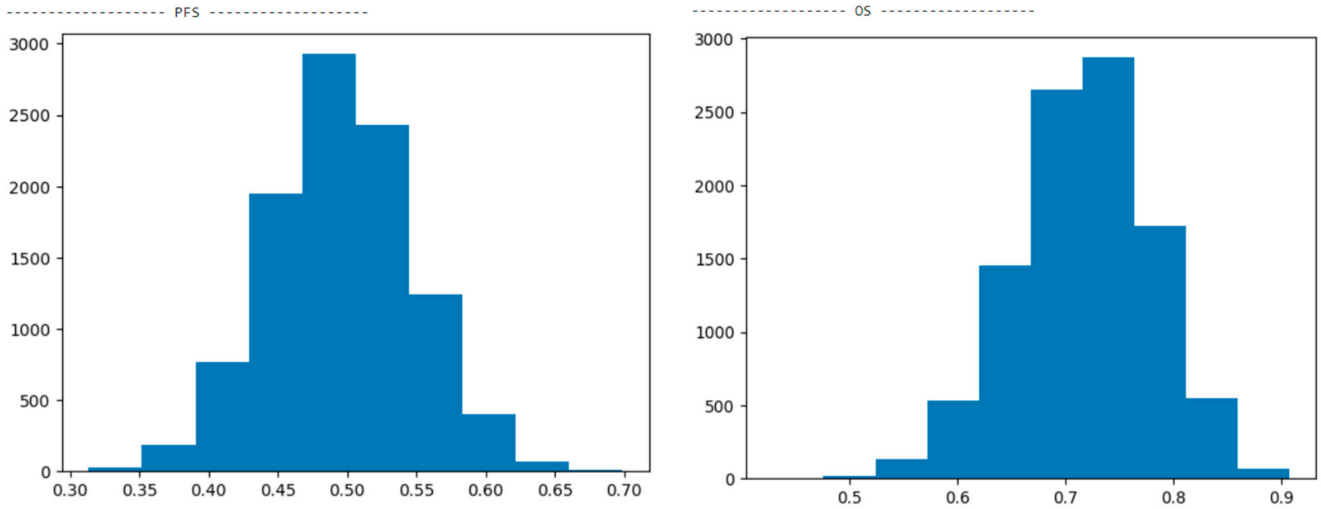

Figure S2 : AUC analysis of TTV for PFS and OS at 6 months

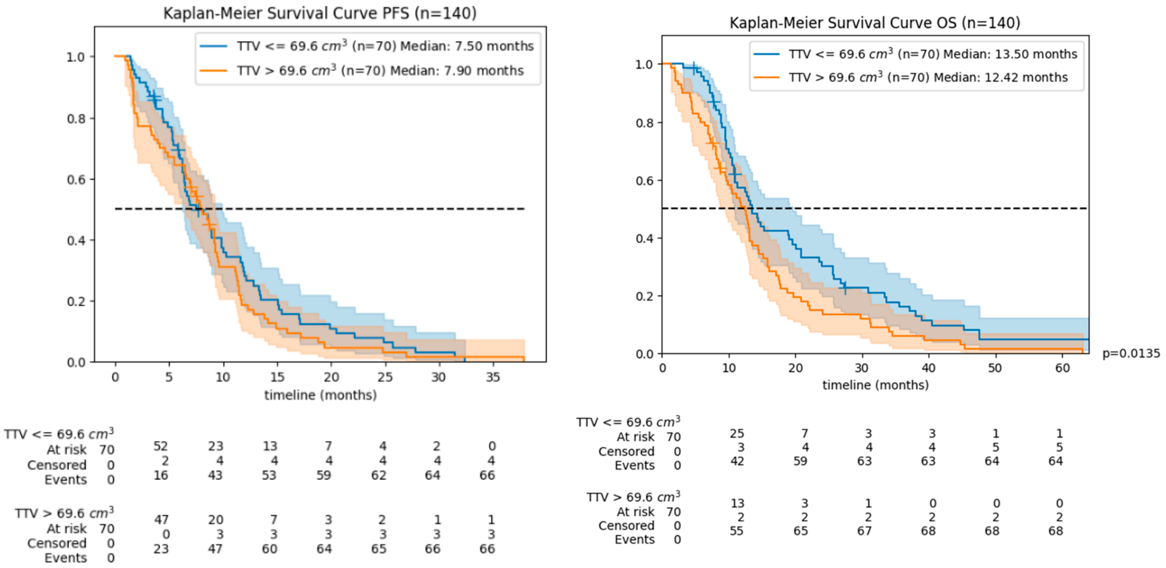

(a) **(b)**

Figure S3 : Kaplan-Meier curves for progression-free survival (**a**) and overall survival (**b**) according to median total tumor volume (TTV).

PFS

covariate	coef	exp(coef)	se(coef)	coef lower 95%	coef upper 95%	exp(coef) lower 95%	exp(coef) upper 95%	cmp to	z	p	-log2(p)
**TTV categorized**	0.471768	1.602826	0.379945	-0.272909	1.216446	0.761162	3.375171	0.0	1.241677	0.214356	2.221920
**Ca19-9 categorized**	0.227751	1.255773	0.225052	-0.213343	0.668845	0.807879	1.951982	0.0	1.011993	0.311541	1.682505
**NLR categorized**	0.888967	2.432615	0.251558	0.395922	1.382012	1.485753	3.982907	0.0	3.533842	0.000410	11.253617

OS

covariate	coef	exp(coef)	se(coef)	coef lower 95%	coef upper 95%	exp(coef) lower 95%	exp(coef) upper 95%	cmp to	z	p	-log2(p)
**TTV categorized**	0.617299	1.853915	0.379994	-0.127476	1.362075	0.880315	3.904285	0.0	1.624497	0.104270	3.261606
**Ca19-9 categorized**	0.411521	1.509111	0.228246	-0.035833	0.858875	0.964801	2.360503	0.0	1.802971	0.071393	3.808079
**NLR categorized**	0.888242	2.430853	0.248275	0.401631	1.374853	1.494260	3.954495	0.0	3.577649	0.000347	11.494033

(a)

------------------- PFS -------------------

covariate	coef	exp(coef)	se(coef)	coef lower 95%	coef upper 95%	exp(coef) lower 95%	exp(coef) upper 95%	cmp to	z	p	-log2(p)
**TTV**	-0.084236	0.919215	0.425828	-0.918844	0.750373	0.398980	2.117789	0.0	-0.197816	0.843189	0.246072
**Ca19-9**	0.304538	1.355998	0.227303	-0.140968	0.750044	0.868517	2.117093	0.0	1.339787	0.180315	2.471411
**NLR**	0.885951	2.425290	0.283143	0.331002	1.440900	1.392363	4.224497	0.0	3.128994	0.001754	9.155087

------------------- OS -------------------

covariate	coef	exp(coef)	se(coef)	coef lower 95%	coef upper 95%	exp(coef) lower 95%	exp(coef) upper 95%	cmp to	z	p	-log2(p)
**TTV**	0.119534	1.126972	0.427033	-0.717435	0.956503	0.488002	2.602581	0.0	0.279918	0.779541	0.359304
**Ca19-9**	0.381192	1.464028	0.226043	-0.061844	0.824228	0.940030	2.280119	0.0	1.686371	0.091724	3.446550
**NLR**	0.801828	2.229612	0.279709	0.253608	1.350047	1.288667	3.857607	0.0	2.866651	0.004148	7.913228

(b)

Table S1 : Univariate **(a)** and multivariate **(b)** analysis on baseline parameters for PFS and OS.
